# A systematic review of the relationship between momentary emotional states and nonsuicidal self‐injurious thoughts and behaviours

**DOI:** 10.1111/papt.12397

**Published:** 2022-05-08

**Authors:** Alexandra C. Brown, Katie Dhingra, Terence D. Brown, Adam N. Danquah, Peter James Taylor

**Affiliations:** ^1^ Division of Psychology & Mental Health Manchester Academic Health Sciences Centre University of Manchester Manchester UK; ^2^ School of Social Sciences Leeds Beckett University Leeds UK; ^3^ The Postgraduate Medical Education Council of Tasmania Hobart Tasmania Australia

**Keywords:** affect, ecological momentary assessment, emotion, experience sampling methodology, non‐suicidal self‐injury, self‐Injury

## Abstract

**Background:**

Nonsuicidal self‐injury (NSSI) is associated with high levels of distress, co‐morbid mental health issues, and elevated risk of suicide. Previous literature indicates that emotion regulation is the most endorsed function of NSSI. Experience Sampling Methodology (ESM) provides a powerful tool for investigating the moment‐to‐moment associations between emotional states and NSSI thoughts and behaviours. The aim of the current study was to systematically review and evaluate ESM research concerning the relationship between momentary emotional states and NSSI.

**Methods:**

A systematic search of electronic databases from date of inception to 16th April 2021 was conducted. This was supplemented through backwards citation tracking. A risk of bias assessment was completed prior to data synthesis.

**Results:**

Nineteen eligible studies were identified for inclusion in the review. Heightened negative affect was found to typically precede instances of NSSIT thoughts and behaviour. Results were less consistent for positive affect.

**Limitations:**

Sample sizes across studies were often small, meaningful effect sizes were not always reported, and non‐validated measures of NSSI thoughts and behaviour were used during ESM assessments.

**Conclusions:**

The results support affect regulation models of NSSI, and demonstrate the value of ESM studies, specifically those sampling more than once per day, in plotting the temporal, “in‐the‐moment” characteristics of these processes.

**Practitioner points**

- ESM can investigate the temporal pattern of emotions in those who self‐injure.
- Negative affect typically increases before and declines following self‐injury; findings are mixed with regards to positive affect and self‐injury.
- ESM studies in this area are limited by small samples and a lack of validated measures of momentary NSSI.
- Interventions that focus on disrupting the connection between momentary changes in affect and NSSI (e.g. developing alternative responses) may be helpful.

## INTRODUCTION

Non‐suicidal self‐injury (NSSI) refers to the intentional, direct injuring of body tissue for a purpose that is not socially sanctioned, without suicidal intent (Klonsky, 2007; Muehlenkamp, 2005). NSSI may include cutting, burning, biting, scratching, preventing wound healing, or banging and hitting various body parts (Klonsky, 2007). It is a prevalent health‐risk issue, with the self‐reported lifetime prevalence of NSSI in those aged 16–74 in England in 2014 estimated at 6.4%, an increase from 3.8% in 2007 (McManus et al., 2016). A meta‐analysis by Swannell et al. (2014) stated that the pooled global lifetime prevalence of NSSI was 17.2% among adolescents, 13.4% among young adults, and 5.5% among adults. NSSI is predictive of later emotional difficulties (Duakantaité et al., 2020), and those who engage in self‐injurious behaviour and experience self‐injurious thoughts are at increased risk of later suicide attempts and death by suicide (Lofthouse & Yager‐Schweller, 2009; Ribiero et al., 2016). NSSI thoughts and urges (NSSIT) can be experienced by individuals who do and do not go on to engage in NSSI, however, they have received less research attention compared to NSSI behaviour (Martin et al., 2011).

NSSI can serve various functions for individuals, such as the communication of needs, self‐punishment, sensation seeking, anti‐dissociation, or a way to resist suicidal urges (Klonsky, 2007; McManus et al., 2016; Taylor et al., 2018). The most commonly endorsed function is emotion regulation, for example, to alleviate negative emotions such as anger or shame (Taylor et al., 2018). Emotion regulation describes the process of how people influence their emotional experience and responses, for example, reducing the intensity or valence of a feeling (McKenzie & Gross, 2014). Across many different theoretical models of NSSI, a common idea is that NSSI occurs in response to distressing emotional states, providing a means of regulating these states by modifying, reducing, or distracting from them in some way (e.g. Chapman et al., 2006; Hasking et al., 2017; Selby & Joiner, 2009). The Emotional Cascade Model, for example, suggests individuals become trapped in an escalating cycle of negative affect and rumination, and that NSSI provides a means of breaking free from this (Selby & Joiner, 2009). It has also been suggested that offset of pain following the end of a self‐injurious act may also alleviate negative feelings in some people (Hooley & Franklin, 2017). It is possible some negative emotions may be more closely linked to NSSI than others. For example, feelings of shame are robustly correlated with the presence and severity of NSSI (Sheehy et al., 2019). It has been hypothesised that for some individuals NSSI may alleviate such feelings by acting as a form of self‐punishment (Hooley & Franklin, 2017; Sheehy et al., 2019). There are differing views on whether individuals who engage in NSSI are more vulnerable to intense negative emotional states (e.g. Hasking et al., 2017; Hooley & Franklin, 2017), but theories generally agree on how the use of NSSI to regulate such feelings can then reinforce the use of NSSI in the future.

Longitudinal studies into NSSI and emotional states can provide information about temporal patterns of association occurring across broad periods of time, but are less able to capture finer, moment‐by‐moment patterns in how emotional states and NSSI interact. Examining moment‐to‐moment data could potentially provide a stronger insight into what may be triggering or maintaining NSSI. Experience Sampling Methodology (ESM; also called Ecological Momentary Assessment [EMA]),^1^ involves the use of multiple daily assessments to track phenomena on a day‐to‐day and moment‐to‐moment basis. ESM has been widely adopted to further our understanding of NSSI and related phenomena (Pratt & Taylor, 2019). The ability to reduce problems of recall bias by asking about experiences as they occur, and the ability to track intra‐individual changes in phenomena, are recognised advantages of ESM (Bolger & Laurenceau, 2013; Palmier‐Claus et al., 2019). It is also noted that statistical power may be increased through the inclusion of a large number of data points per person, though proper sample size justification remains as important as for any research (Bolger & Laurenceau, 2013).

ESM studies allow the separation of state negative affect (i.e. brief, in‐the‐moment periods of negative feeling) from trait negative affect (i.e. a general predisposition to experience negative affect). Other psychological processes that are putatively linked to the experience of affective states, such as rumination, a process of repetitive negative thinking, can also be studied at the state level with ESM. Whilst ESM has been used to study the frequency and intensity of affective states, it can also be used to estimate instability in such states (i.e. how much affective states change or vary over a short period of time; Palmier‐Claus, Taylor, Gooding, et al., 2012). This is relevant given that instability in affect may increase the risk of self‐injurious behaviour, above and beyond the relative intensity of feeling, through the sensitisation of emotion‐linked, self‐injury related cognitions and urges (e.g. Palmier‐Claus, Taylor, Varese et al., 2012; Selby et al., 2013).

The current paper aims to provide a systematic review of current ESM literature that examines relationships between momentary emotional states both prior to and following NSSI and NSSIT. The temporal characteristics regarding the relationship between NSSI, NSSIT and emotional states will be considered. A greater understanding of these processes will help further validate models of NSSI and inform interventions. A recent review (Rodríguez‐Blanco et al., 2018) provides an overview of ESM studies focused NSSI studies, reporting that negative emotions tend to be elevated prior to NSSI. However, they did not focus on emotional states, specifically, and only examine these effects in limited detail. Details regarding the types of models and associations tested and the magnitude of effects are lacking, as well as a formal evaluation of the risk of bias. Moreover, a number of relevant papers have since been published in this area. The present review, despite its narrower focus, also includes additional studies (*n* = 10). Given this rapidly evolving literature, an updated review focused specifically on the ESM studies concerning the link between momentary emotional states and NSSI is warranted.

## MATERIALS AND METHODS

### Registration and reporting

This review follows the Preferred Reporting Items for Systematic Reviews and Meta‐Analyses (PRISMA; Moher et al., 2009) guidelines. A review protocol was pre‐registered (PROSPERO ID CRD42019137093). There was one departure from the original protocol: (1) initially the review was going to encompass suicidal behaviours, but the focus was later revised to be NSSI specifically (see Appendix S1 for further details).

### Search strategy

Electronic databases PsycInfo, MEDLINE, CINAHL and Web of Science were systematically searched from date of inception to 16th April 2021 using the following search terms and Boolean operators: (self‐injur* OR self‐injurious OR NSSI OR self‐harm* OR DSH OR self‐mutilat* OR overdos* OR self‐poison* OR self‐cut* OR suicid*) AND (emotion* OR feeling* OR affect* OR distress OR anger OR shame OR sadness) AND (ESM OR EMA OR “experience sampl*” OR diar* OR momentary). The search terms were selected based on scoping searches and knowledge of the literature. For example, studies often refer to NSSI or self‐harm, or more rarely to self‐cutting, but studies do not appear to refer to “self‐hitting” without also using these other terms. Medical subject headings that mapped onto keywords were also included in searches (see Appendix S1 for details).

The reference lists of included studies (backwards tracking), and articles that cited included studies (forwards tracking), were examined to identify any potentially eligible papers not identified in the original database search. Authors of included studies were also contacted by the researcher (where contact details were available) in order to request any unpublished data or studies that may be relevant to the review.

Eligibility criteria for inclusion were as follows: studies (a) employed ESM, defined here as completing more than one assessment or sampling per day outside of a laboratory; (b) measured emotional states or experiences as part of the ESM assessments; (c) measured NSSI and/or NSSIT either at momentary or person‐level; (d) included an analysis of the association between emotional states or experiences and NSSI and/or NSSIT (or this was obtained from study authors); (e) were written in English; and (f) included adolescent and/or adult populations. Studies were not included if they used a solely qualitative methodology. Studies that aggregated assessments of suicidal and non‐suicidal phenomena, or where this was unclear, were excluded. NSSIT could encompass both thoughts (i.e. cognitions about NSSI) or urges (i.e. an experienced pressure to engage in NSSI). Within the review, we clarify whether urges or thoughts were the outcome. We excluded daily diary studies that use a single assessment per day. A key feature of the ESM design is the capture of “in‐the‐moment” data on current or very recent thoughts, feelings, and experiences (Palmier‐Claus et al., 2019). In this way, ESM differs to studies using a daily diary approach, where experiences from across the day are usually captured.

### Screening and data extraction

Articles were screened for eligibility at the title and abstract level, and the full text of potentially relevant papers were then screened for eligibility. All articles were screened independently by two members of the research team. Any discrepancies were resolved through discussion within the review team.

Data extraction was completed by the first author using an extraction spreadsheet, and this was then reviewed by the wider research team. Information extracted included basic information (e.g. year published, country of origin, source, study design, sampling method), participant characteristics (e.g. sample size, demographic information, specific populations), method of and measures used for ESM, measures of emotion and NSSI/NSSIT during ESM, and whether further information, such as clarification or unpublished data, had been sought from authors.

Given the expected broad range of different emotional states being studied, the variation in ESM designs, and how data analysis was approached, a meta‐analysis was not planned.

### Risk of bias assessment

Risk of bias was assessed using a tool adapted from the Agency for Healthcare Research and Quality (Williams et al., 2010; see Appendix S1 for the adapted version used in this review and further details of development). This tool has been adapted for use in previous reviews on the topic of NSSI (Taylor et al., 2015; Taylor et al., 2018). In this review, the researchers adapted the tool by creating three additional domains focusing on aspects of ESM design: time‐stamped ESM responses (i.e. assessments were completed via a method that ensures time of completion is recorded), pseudo‐random prompts (i.e. prompts were delivered at random points within set time intervals), and timepoints completed within 15 min of prompts.

Each study was rated independently by two members of the research team. Domains were rated as being met (low risk of bias), not met (high risk), partially met, or being unclear. The two reviewers assessed all included articles and discussed and resolved any discrepancies.

## RESULTS

### Overview of studies

**FIGURE 1:**
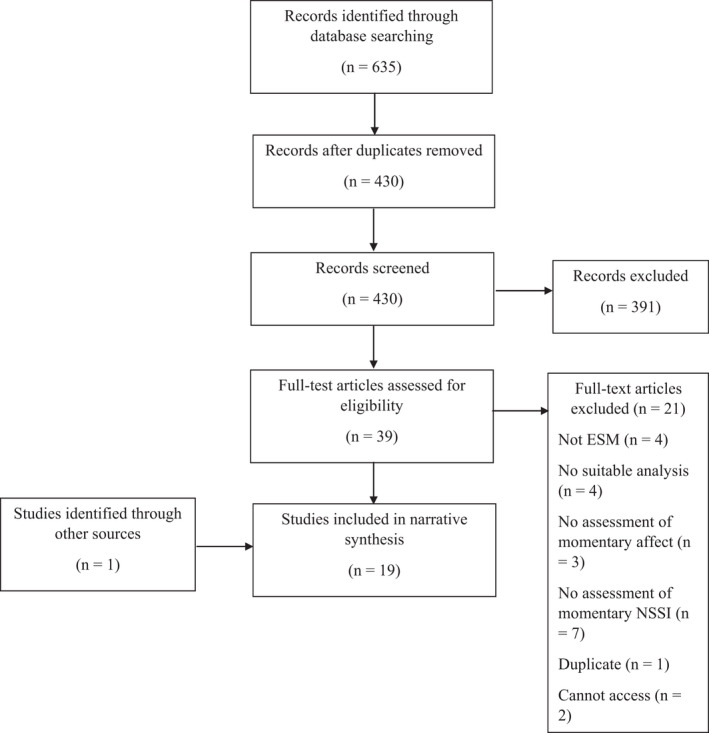
Flow chart of literature search process

The full search process can be seen in Figure 1. Nineteen eligible papers were identified for inclusion in this review. Characteristics of these studies are summarised in Table 1.

**TABLE 1:** Study characteristics

Author, year & country	Sample characteristics	ESM design (*n*. scheduled prompts)	Compliance	Emotional state (measure)	NSSI/NSSIT (measure)
Ammerman et al. (2017), USA	Current diagnosis of BPD and depressive disorder (*n* = 51), 74.5% female, mean age = 28.8 (*SD =* 9.8), 51% African American, 33% White, 10% Asian, 6% “other”	Phone calls, four calls per day for 7 days (28)	*M* days where at least one assessment completed = 5.5 (of 7)	Negative affect (PANAS)	Non‐validated measure of NSSI occurrence
Andrewes et al. (2017a), Australia	15–25 year olds with first presentation BPD (*n* = 107), 82.3% female and mean age = 18.1 (*SD* = 2.7), 91% Caucasian, 2.8% Indigenous Australian, remainder not reported	Smartphone, six prompts per day for 6 days (36)	52% of entries completed	Negative and positive affect (PANAS‐SF)	Non‐validated measure of NSSI occurrence
Andrewes et al. (2017b), Australia	15–25 year olds with first presentation BPD (*n* = 107), 82.3% female and mean age = 18.1 (*SD* = 2.7), 91% Caucasian, 2.8% Indigenous Australian, remainder not reported	Smartphone, six prompts per day for 6 days (36)	52% of entries completed	Negative affect (PANAS‐SF)	Non‐validated measure of NSSI occurrence
Anestis et al. (2012), USA	Female patients with BN (*n* = 127), mean age = 25.3 (*SD* = 7.7), 96.9% Caucasian, 1.5% Native American, 0.8% Asian, 0.8% “other”	Palm‐top computer, three prompts per day for 14 days (42)	Not reported	Negative affect (PANAS)	Non‐validated measure of NSSI occurrence based on various validated measures (e.g., Rossotto et al., 1998)
Armey et al. (2011), USA	Students with a NSSI history (*n* = 36), 75% female, mean age = 18.7 (*SD* = 0.8), ethnicity data unavailable but states “predominantly Caucasian”	Palm‐top computer, six prompts per day for 7 days (42)	38% of entries completed	Negative affect (PANAS; and PANAS‐X guilt and hostility subscales)	Non‐validated measure of NSSI occurrence
Briones‐Buixassa et al. (2021), Spain	University students who reported self‐injuring ≥5 times in the past year (*n* = 19), mean age = 19.7 (*SD* = 1.6), ethnicity data not reported^a^ Patients with BPD diagnosis who reported self‐injuring ≥5 times in the past year (*n* = 22), mean age = 23.6 (*SD* = 5.1), ethnicity data not reported^a^ Adults without any past or current mental health disorders (*n* = 23), mean age = 22.7 (*SD* = 4.4), ethnicity data not reported^a^	Smartphone, three prompts per day for 15 days (45), NSSI‐related event‐contingent entries also allowed	80% of entries completed	Anger, sadness, guilt, and frustration (no validated measures)	Non‐validated measure of NSSI occurrence
Dillon et al. (2021), USA	Veterans with NSSI disorder diagnosis (*n* = 40), 28% female, mean age = 46.7 (*SD* = 12.8), 55% Black, 45% White	Smartphone, three prompts per day for 28 days (84), NSSI‐related event‐contingent entries also allowed	82% of entries completed	Anger/hostility (PANAS‐X)	Non‐validated measure of NSSI occurrence; non‐validated measure of NSSIT occurrence
Hepp et al. (2021), USA	Adults with BPD diagnosis (*n* = 56), 82% female, mean age = 26.0 (*SD* = 7.2), 84% Caucasian	Palm‐top computer, average of six prompts per day for approximately 21 days (126), plus entries made every morning, and NSSI or drinking‐related event‐contingent entries allowed	*M* of 89% of entries (in response to random prompts) completed per person	Negative affect (PANAS‐X)	Non‐validated measure of NSSIT occurrence
Houben et al. (2017), Belgium	Inpatients with BPD diagnosis (*n* = 30), 87% female, mean age = 29.0 (*SD* = 1.6), ethnicity data not reported	Palm‐top computer, 10 prompts per day for 8 days (80)	*M* of 66% of entries completed per person	Negative and positive affect (no validated measures)	Non‐validated measure of NSSI occurrence
Hughes et al. (2019), USA	Adolescents and young adults who reported self‐injuring ≥2 times during previous 2 weeks (*n* = 47), 68% female, mean age = 19.1 (*SD* = 1.8), 38% White, 15% Black/African American, 19% Asian, 17% Hispanic/Latino, 11% Multi‐Racial	Smartphone, five prompts per day for 14 days (70), NSSI‐related event‐contingent entries also allowed	*M* of 71 entries competed per person	Negative affect (no validated measures)	Non‐validated measure of frequency of NSSI/NSSIT
Kiekens et al. (2020), Belgium	University students who reported self‐injuring ≥5 days during previous year and reported urges to self‐injure in past month (*n* = 30), 80% female, Mean age = 20.1 (*SD* = 1.1), ethnicity data not reported	Smartphone, eight prompts per day for 12 days (96)	Median of 79% of entries completed per person	Negative and positive affect (no validated measures)	Non‐validated measure of frequency of NSSI; non‐validated measure of intensity of NSSIT
Kranzler (2016), USA	Adolescents and young adults who reported self‐injuring ≥2 times during previous 2 weeks (*n =* 24), 66.7% female, mean age = 19.3 (*SD* = 1.8), 45.8% White, 8.3% African American, 20.8% Asian, 12.5% Hispanic/Latino, 12.5% Multi‐Racial	Smartphone, five prompts per day for 14 days (70), NSSI‐related event‐contingent entries also allowed	All completed ESM protocol and 22 (92%) completed ≥80% of signal contingent entries	Negative and positive affect (no validated measures)	Non‐validated measure of frequency of NSSI; non‐validated measure of intensity of NSSIT
Kranzler et al. (2018), USA	Adolescents and young adults who reported self‐injuring ≥2 times during previous 2 weeks (*n* = 47), 68.1% female, mean age = 19.1 (*SD* = 1.8), 38.3% White, 14.9% African American, 19.1% Asian, 17% Hispanic/Latino, 10.6% Multi‐Racial	Smartphone, five prompts per day for 14 days (70), NSSI‐related event‐contingent entries also allowed	All completed ESM protocol and 40 (85.1%) completed ≥80% of signal contingent entries	Negative and positive affect (no validated measures)	Non‐validated measure of frequency of NSSI; non‐validated measure of intensity of NSSIT
Muehlenkamp et al. (2009), USA	Females with BN diagnosis (*n* = 131), mean age = 25.3 (*SD* = 7.6), 96.9% Caucasian, remainder not reported	Palmtop computer, six prompts per day for 14 days (84), NSSI‐related event‐contingent entries also allowed	Not reported	Negative and positive affect (subset of PANAS items)	Non‐validated measure of NSSI occurrence based on various validated measures (e.g., Rossotto et al., 1998)
Selby et al. (2013), USA	Undergraduate students and community‐based adults reporting ≥4 dysregulated behaviours over previous 2 weeks (*n* = 47), 66% female, mean age unclear, 19% African American, 6% Asian American, 2% Native American, 9% Hispanic, remainder not reported	Palm‐top computer, five prompts per day for 14 days (70)	*M* of 61 entries competed per person (out of 70). Overall compliance >90%	Negative affect (subset of PANAS items)	Frequency of NSSI (no validated measures)
Snir et al. (2015), USA	Adults with APD diagnosis (*n* = 43, 53.5% female, mean age = 32.9, *SD* = 11.4), BPD diagnosis (*n* = 56, 80.4% female, mean age = 30.9, *SD* = 10.1), and healthy controls (*n* = 53, 71.7% female, mean age = 35.08, *SD* = 11.9), 7.1% Asian, 19.6% Black/African, 60.7% White, 14.2% “other”	Palm‐top computer, five prompts per day for 21 days (105)	*M* of 74 entries competed per person (out of 105)	Negative affect (no validated measures)	Occurrence of NSSI and NSSIT (no validated measures)
Vansteelandt et al. (2017), Belgium	Adults with BPD diagnosis (*n* = 32), 84% female, mean age = 28 (*SD* = 9), ethnicity data not reported	Palm‐top computer, 10 prompts per day for 8 days (80)	*M* of 50 entries competed per person (out of 80)	Emotional valence and activation (no validated measures)	Frequency of NSSI (no validated measures)
Victor et al. (2019), USA	Women taking part in Pittsburgh Girls Study who endorsed self‐injurious urges in previous month (*n* = 62), mean age = 22.0 (*SD* = 1.6), 71% African American, 24.2% non‐Hispanic Caucasian, 1.6% Hispanic African American, 4.8% Multiracial/biracial	Smartphone, six prompts per day for 21 days (147)	*M* of 110 entries per person (out of 147). *N* = 29 (46.8%) achieved 85% compliance; over two thirds (n = 43, 69.4%) completed at least 70% of expected entries, and 80.6% (*n* = 50) completed at least 50% of expected entries	Negative affect (no validated measures)	Non‐validated measure of NSSIT occurrence
Zaki et al. (2013), USA	Adults with BPD diagnosis and history of NSSI (*n* = 38, 84% female, mean age = 29.89, *SD* = 10.60) and healthy controls (*n* = 42, 83% female, mean age = 32.50, *SD* = 7.53), 61% White, 18% Black/African, 8% Asian, 18% Hispanic, 5% “other”	Electronic technology‐ not specified, five prompts per day for 21 days (105)	*M* of 76 entries per person (out of 105)	Negative affect (no validated measures)	Non‐validated measure of NSSI and NSSIT frequency (no validated measures)

Abbreviations: Borderline personality disorder (BPD), bulimia nervosa (BN), avoidant personality disorder (APD). PANAS (Watson et al., 1988); PANAS‐SF (Kercher, 1992); PANAS‐X (Watson & Clark, 1994).

^a^ Gender is reported only as a percentage but these values to do not match up with the report sample size for groups and hence have been excluded from this data.

Studies most commonly took place in the USA (*k* = 13), but also in Australia (*k* = 2), Belgium (*k* = 3) and Spain (*k* = 1). There was a mixture of clinical (individuals with a diagnosis of borderline personality disorder (BPD), avoidant personality disorder (APD), bulimia nervosa (BN) or depressive disorders, and community and inpatient mental health patients) and non‐clinical samples (students and members of the community) included in the studies. Three pairs of studies used the same or an overlapping sample (Andrewes et al., 2017a; Andrewes et al., 2017b; Houben et al., 2017; Hughes et al., 2019; Kranzler et al., 2018; Vansteelandt et al., 2017, respectively). It was unclear whether there was overlap in sample between Kranzler (2016) and Kranzler et al. (2018); the samples were very similar, though Kranzler et al. (2018) had a larger sample size than Kranzler (2016). The author was contacted for clarification but did not respond.

The length of the ESM period varied between 6 and 28 days (*M* = 14.0), with between 3 and 10 assessments per day (*M* = 5.6). Seventeen studies investigated general negative affect, six investigated positive affect and nine investigated specific emotions as well. Seventeen studies assessed NSSI behaviour, whilst nine assessed NSSIT (three of these focused on thoughts about NSSI and three on NSSI urges).

### Risk of bias

Formal risk of bias assessment is considered best practice within systematic reviews (e.g. Moher et al., 2009), ensuring that the synthesis of study findings is balanced against a recognition of the weaknesses and potential for bias within the literature. Table 2 shows the results of the risk of bias assessment.

**TABLE 2:** Risk of bias assessment

Study	Unbiased selection of the cohort	Sample size calculated	Adequate description of the cohort	Validated method for ascertaining clinical status or participant group	Validated methods for assessing emotion	Validated methods for assessing NSSI/T	Missing data minimal	Analysis controls for confounding variables	Analytic methods appropriate	Time‐stamped response	Pseudo‐random prompts	Exclude timepoints completed outside 15 minutes
Ammerman et al. (2017)	Unclear	No	Yes	Yes	Yes	No	No	No	Yes	Yes	Yes	Unclear
Andrewes et al. (2017a)	Yes	No	Yes	Yes	Yes	No	No	No	Yes	Yes	Yes	Yes
Andrewes et al. (2017b)	Yes	No	Yes	Yes	Yes	No	No	No	Yes	Yes	Yes	Yes
Anestis et al. (2012)	No	No	Yes	Yes	Yes	Partial	Unclear	Yes	Yes	Yes	Yes	Unclear
Armey et al. (2011)	Unclear	No	Partial	Yes	Yes	No	No	No	Yes	Yes	Yes	Yes
Briones‐Buixassa et al. (2021)	No	Unclear	Yes	Yes	No	No	Yes	Yes	Yes	Yes	Yes	Yes
Dillon et al. (2021)	Partial	No	Yes	Yes	Yes	No	Yes	No	Yes	Yes	Yes	No
Hepp et al. (2021)	No	No	Yes	Yes	Yes	No	Yes	Yes	Yes	Yes	Yes	Yes
Houben et al. (2017)	Unclear	No	Partial	Yes	No	No	Partial	No	Partial	Yes	Yes	Unclear
Hughes et al. (2019)	No	No	Yes	Yes	No	No	Yes	Yes	Partial	Yes	Yes	No
Kiekens et al. (2020)	Yes	No	Partial	Yes	Partial	No	Yes	Yes	Yes	Yes	Yes	Yes
Kranzler (2016)	No	No	Yes	Yes	No	No	Yes	Yes	Yes	Yes	Yes	No
Kranzler et al. (2018)	No	No	Yes	Yes	No	No	Yes	Yes	Yes	Yes	Yes	No
Muehlenkamp et al. (2009)	Unclear	No	Yes	Yes	Yes	Partial	Unclear	No	Yes	Yes	Yes	Unclear
Selby et al. (2013)	Partial	No	Yes	No	Partial	No	Yes	Yes	Yes	Yes	Yes	Unclear
Snir et al. (2015)	No	No	Yes	Yes	No	No	Unclear	No	Yes	Yes	Yes	Unclear
Vansteelandt et al. (2017)	Unclear	No	Partial	Yes	No	No	Partial	Yes	Yes	Yes	Yes	Unclear
Victor et al. (2019)	Yes	No	Yes	Yes	Partial	No	Yes	Yes	Yes	Yes	Yes	No
Zaki et al. (2013)	No	No	Yes	Yes	No	No	Unclear	Yes	Yes	Yes	Yes	Unclear

No studies clearly justified sample size. Fourteen studies had sample sizes of less than 100, with one study only having *n* = 24 (Kranzler, 2016), and some studies also undertaking analyses on small subsamples of their data, increasing the risk of possible Type II errors. Many studies relied heavily on sampling methods that could amplify self‐selection bias, such as adverts. Another recurrent issue was the lack of validated assessment methods used during ESM, especially for NSSI.

Compliance with ESM data entry was generally good, though six papers had missing data exceeding 70%. While statistical models can often reduce any bias where data are Missing at Random (MAR; Carter & Emsley, 2019), there is still information loss in these circumstances, and bias may still be introduced if missing data are not MAR. All studies met the criteria for pseudorandom ESM prompts, and the majority of papers also met the criteria for the use of validated methods in ascertaining clinical status or participant group. Papers often employed multi‐level regression, which is an appropriate analytic method for nested ESM data. However, two papers examining lagged effects attempted to control for the outcome at previous assessment points, including this as a covariate, which violates the assumption that covariates in the models are independent of random effects (Carter & Emsley, 2019). All papers used electronic diary methods that ensure a timestamp, such as smartphone applications or palm pilots, except one study that used telephone calls. It was sometimes unclear whether ESM entries outside of a 15‐minute response window were removed from analyses, as is recommended so that responses reflect “in the moment” experience (Palmier‐Claus et al., 2011).

### Synthesis of results

The results are summarised below. We have grouped these based on whether NSSIT or NSSI was the outcome and based on the type of association or effect being investigated. Table 3 provides a further summary of concurrent lagged associations tested within these studies. Several papers examined more complex non‐linear effects that cannot be readily described with a single statistic, and these have been left out of Table 3, but are described below. The majority of analyses used mixed models that accounted for both within and between participant effects. Three studies aggregated variables across the ESM period allowing for only between‐person differences to be investigated.

**TABLE 3:** Summary of key study effect sizes

Author (year)	Predictor	Outcome	Analysis	Unadjusted effect	Adjusted effect
Ammerman et al. (2017)	Negative affect (range: 0–40, greater values mean greater negative affect)	Daily NSSI (binary)	Mixed‐model, concurrent association	*B* = 5.46*	None
Anestis et al. (2012)	Negative affect (average across ESM period)	Number of NSSI episodes	Between‐person, concurrent association	*r* = .04, n.s.	*r*_part_ = .02, n.s. (adjusting for: affect instability, depression, disordered eating, suicide attempt history, interaction of suicide attempt history and affective lability)
Anestis et al. (2012)	Affective instability (across ESM period)	Number of NSSI episodes	Between‐participants, concurrent association	*r* = .17, n.s.	*r*_part_ = .01, n.s. (adjusting for: negative affect, depression, disordered eating, suicide attempt history, interaction of suicide attempt history and affective lability)
Briones‐Buixassa et al. (2021)	Anger (range: 0–100, greater values mean greater anger)	NSSI (binary)	Between‐person, concurrent association	None	*OR* = 0.92* (adjusting for: other negative affect variables, person‐level affective problems, Borderline Personality symptoms, metacognitive skill). Several significant interactions are reported
Briones‐Buixassa et al. (2021)	Sadness (range: 0–100, greater values mean greater sadness)	NSSI (binary)	Between‐person, concurrent association	None	*OR* = 0.98, n.s. (adjusting for: other negative affect variables, person‐level affective problems, Borderline Personality symptoms, metacognitive skill). Several significant interactions are reported
Briones‐Buixassa et al. (2021)	Guilt (range: 0–100, greater values mean greater guilt)	NSSI (binary)	Between‐person, concurrent association	None	*OR* = 0.98, n.s. (adjusting for: other negative affect variables, person‐level affective problems, Borderline Personality symptoms, metacognitive skill). Several significant interactions are reported
Briones‐Buixassa et al. (2021)	Frustration (range: 0–100, greater values mean greater frustration)	NSSI (binary)	Between‐person, concurrent association	None	*OR* = 1.05* (adjusting for: other negative affect variables, person‐level affective problems, Borderline Personality symptoms, metacognitive skill). Several significant interactions are reported
Dillon et al. (2021)	Anger/hostility (range: 0–4, greater values mean greater anger/hostility)	NSSIT (binary)	Within‐person, lagged association	*B* = 0.42*	None
Dillon et al. (2021)	Anger/hostility (range: 0–4, greater values mean greater anger/hostility)	NSSI (binary)	Within‐person, lagged association	*B* = 0.32*	None
Hepp et al. (2021)	Negative affect (range: 1–5, greater values mean greater negative affect)	NSSIT (binary)	Within‐person, concurrent association	None	OR = 7.50* (adjusting for a large number of covariates relating to the affective, interpersonal and environmental context)
Hepp et al. (2021)	Negative affect (range: 1–5, greater values mean greater negative affect)	NSSIT (binary)	Between‐person, concurrent association	None	OR = 5.00* (adjusting for a large number of covariates relating to the affective, interpersonal and environmental context)
Hepp et al. (2021)	Negative affect (range: 1–5, greater values mean greater negative affect)	NSSIT (binary)	Within‐person, lagged association	None	OR = 2.38, n.s. (adjusting for a large number of covariates relating to the affective, interpersonal and environmental context)
Houben et al. (2017)	Negative affect (range: 0–100, greater values mean greater negative affect)	NSSI (binary)	Mixed‐model, lagged association	*B* = 0.03*	None
Houben et al. (2017)	Positive affect (range: 0–100, greater values mean greater positive affect)	NSSI (binary)	Mixed‐model, lagged association	*B* = −0.01, n.s.	None
Hughes et al. (2019)	Negative affect (range: 0–110, greater value means greater negative affect)	NSSIT (range: 0–10, greater value means more intense thoughts)	Mixed‐model, lagged association	*B* = 0.01, RR = 1.01^a^ ^,^ *	*B* = 0.01, RR = 1.01^a^ ^,^ * (adjusting for repetitive negative thinking and its interaction with negative affect)
Hughes et al. (2019)	Anxiety (range: 0–10, greater value means greater negative affect)	NSSIT (range: 0–10, greater value means more intense thoughts)	Mixed‐model, lagged association	*B* = 0.05, RR = 1.05^a^ ^,^ *	*B* = 0.04, RR = 1.04^a^ ^,^ * (adjusting for repetitive negative thinking and its interaction with negative affect)
Hughes et al. (2019)	Feeling overwhelmed (range: 0–10, greater value means greater negative affect)	NSSIT (range: 0–10, greater value means more intense thoughts)	Mixed‐model, lagged association	*B* = 0.05, RR = 1.05^a^ ^,^ *	*B* = −0.01, RR = 0.99,^a^ n.s. (adjusting for gender, repetitive negative thinking and its interaction with negative affect)
Hughes et al. (2019)	Negative affect (range: 0–110, greater value means greater negative affect)	NSSI frequency	Mixed‐model, lagged association	*B* = 0.03, RR = 1.03^a^ ^,^ *	*B* = 0.02, RR = 1.02^a^ ^,^ * (adjusting for repetitive negative thinking and its interaction with negative affect)
Hughes et al. (2019)	Anxiety (range: 0–10, greater value means greater negative affect)	NSSI frequency	Mixed‐model, lagged association	*B* = 0.07, RR = 1.08^a^ ^,^ *	*B* = 0.02, RR = 1.02,^a^ n.s. (adjusting for repetitive negative thinking and its interaction with negative affect)
Hughes et al. (2019)	Feeling overwhelmed (range: 0–10, greater value means greater negative affect)	NSSI frequency	Mixed‐model, lagged association	*B* = 0.08, RR = 1.09^a^ ^,^ *	*B* = −0.01, RR = 0.99,^a^ n.s. (adjusting for repetitive negative thinking and its interaction with negative affect)
Kiekens et al. (2020)	Negative affect (range: 0–6, greater value means greater negative affect)	NSSIT (range: 0–6, greater value means more intense thoughts)	Mixed‐model, concurrent associations	*B* = 0.48 (credibility interval: 0.43, 0.53)^b^	*B* = 0.28 (credibility interval: 0.22, 0.33)^b^ (adjusting for: positive affect, self‐efficacy to resist NSSI)
Kiekens et al. (2020)	Positive affect (range: 0–6, greater value means greater negative affect)	NSSIT (range: 0–6, greater value means more intense thoughts)	Mixed‐model, concurrent associations	*B* = −0.33 (credibility interval: −0.37, −0.29)^b^	*B* = −0.05 (credibility interval: −0.09, −0.00)^b^ (adjusting for: negative affect, self‐efficacy to resist NSSI)
Kiekens et al. (2020)	Negative affect (range: 0–6, greater value means greater negative affect)	NSSIT (range: 0–6, greater value means more intense thoughts)	Within‐person, lagged associations	*B* = 0.17 (credibility interval: 0.11, 0.23)^b^	*B* = 0.09 (credibility interval: 0.02, 0.17)^b^ (adjusting for: positive affect, self‐efficacy to resist NSSI)
Kiekens et al. (2020)	Positive affect (range: 0–6, greater value means greater negative affect)	NSSIT (range: 0–6, greater value means more intense thoughts)	Within‐person, lagged associations	*B* = −0.10 (credibility interval: −0.15, −0.05)^b^	*B* = 0.00 (credibility interval: −0.05, 0.06)^b^ (adjusting for: negative affect, self‐efficacy to resist NSSI)
Kiekens et al. (2020)	Negative affect (range: 0–6, greater value means greater negative affect)	NSSI (binary)	Within‐person, lagged associations	*B* = 0.26 (credibility interval: 0.12, 0.41)^b^	None
Kiekens et al. (2020)	Positive affect (range: 0–6, greater value means greater negative affect)	NSSI (binary)	Within‐person, lagged associations	*B* = −0.19 (credibility interval: −0.32, −0.09)^b^	None
Kranzler (2016)	Negative affect (range: 0–130, greater value means greater negative affect)	NSSIT (range: 0–10, greater value means more intense thoughts)	Mixed‐model, lagged association	*B* = 0.02, RR = 1.02*	*B* = 0.02, RR = 1.02* (adjusting for positive affect, age, gender, person‐level positive and negative affect, lifetime NSSI frequency)
Kranzler (2016)	Positive affect (range: 0–80, greater value means greater positive affect)	NSSIT (range: 0–10, greater value means more intense thoughts)	Mixed‐model, lagged association	*B* = −0.02, RR = 0.98*	*B* = −0.01, RR = 0.99* (adjusting for negative affect, age, gender, person‐level positive and negative affect, lifetime NSSI frequency)
Kranzler (2016)	Negative affect (range: 0–130, greater value means greater negative affect)	NSSI frequency	Mixed‐model, lagged association	*B* = 0.01, RR = 1.01*	*B* = 0.04, RR = 1.04* (adjusting for positive affect, age, gender, person‐level positive and negative affect, lifetime NSSI frequency)
Kranzler (2016)	Positive affect (range: 0–80, greater value means greater negative affect)	NSSI frequency	Mixed‐model, lagged association	*B* = 0.00, RR = 1.00, n.s.	*B* = 0.01, RR = 1.01, n.s. (adjusting for negative affect, age, gender, person‐level positive and negative affect, lifetime NSSI frequency)
Kranzler et al. (2018)	Negative affect (range: 0–130, greater value means greater negative affect)	NSSIT (range: 0–10, greater value means more intense thoughts)	Mixed‐model, lagged association	None	*B* = 0.02, RR = 1.02* (adjusting for: momentary positive affect, trait positive and negative affect, lifetime NSSI, depression and BPD symptoms)
Kranzler et al. (2018)	Positive affect (range: 0–80, greater value means greater positive affect)	NSSIT (range: 0–10, greater value means more intense thoughts)	Mixed‐model, lagged association	None	*B* = −0.01, RR = 0.99* (adjusting for: momentary negative affect, trait positive and negative affect, lifetime NSSI, depression and BPD symptoms)
Kranzler et al. (2018)	Negative affect (range: 0–130, greater value means greater negative affect)	NSSI frequency	Mixed‐model, lagged association	None	*B* = 0.03, RR = 1.03* (adjusting for: momentary positive affect, trait positive and negative affect, lifetime NSSI, depression and BPD symptoms)
Kranzler et al. (2018)	Negative affect (range: 0–130, greater value means greater negative affect)	NSSI frequency	Mixed‐model, lagged association	None	*B* = −0.00, n.s. (adjusting for: momentary positive affect, trait positive and negative affect, lifetime NSSI, depression and BPD symptoms)
Selby et al. (2013)	Affective instability (across ESM period)	NSSI events	Between‐person, concurrent association	None	*B* = 0.91* (adjusting for rumination instability; also a significant interaction between rumination instability and affective instability)
Victor et al. (2019)	Internalising negative affect (range: 1–5, greater values means greater negative affect)	NSSI urges (binary)	Within‐person, lagged	None	*β* = .24 (credibility interval: .15, .33)^b^ (adjusting for drug use, alcohol use, externalising negative affect, past‐month NSSI urges, depression and BPD symptoms)
Victor et al. (2019)	Average internalising negative affect (across ESM period)	NSSI urges (binary)	Between‐person, lagged	None	*β* = .49 (credibility interval: −.05, .92)^b^ (adjusting for drug use, alcohol use, externalising negative affect, past‐month NSSI urges, depression and BPD symptoms)
Victor et al. (2019)	Externalising negative affect (range: 1–5, greater values means greater externalising negative affect)	NSSI urges (binary)	Within‐person, lagged	None	*β* = .01 (credibility interval: −.11, .12)^b^ (adjusting for drug use, alcohol use, internalising negative affect, past‐month NSSI urges, depression and BPD symptoms)
Victor et al. (2019)	Average externalising negative affect (across ESM period)	NSSI urges (binary)	Between‐person, lagged	None	*β* = −.42 (credibility interval: −.90, .14)^b^ (adjusting for drug use, alcohol use, internalising negative affect, past‐month NSSI urges, depression and BPD symptoms)
Zaki et al. (2013)	Negative emotion differentiation (aggregate from across ESM period)	Average NSSI acts and urges (across ESM period)	Between‐person, concurrent	None	*B* = −0.41, *β* = −.16 (adjusting for rumination, number of diary entries; also a significant interaction between rumination and negative emotion differentiation)

*Note*: *β* = standardised coefficient; *B* non‐standardised coefficient. *RR* = Relative risk ratio.

^a^ It is not clear from the paper whether these regression coefficients are standardised or unstandardised in nature.

^b^ This analysis used Bayesian inference with credibility intervals. Credibility intervals that do not cross zero are considered to indicate meaningful effects.

* *p* < .05; n.s. = *p* < .05.

### Nonsuicidal Self‐Injurious thoughts (NSSIT)

#### Concurrent associations of affect with NSSIT

Two studies examined the concurrent association between negative affect and NSSI urges (Hepp et al., 2021) and thoughts (Kiekens et al., 2020). One of these adjusted for a large number of contextual factors relating to the environment, emotions, and interpersonal factors (Hepp et al., 2021). In both studies, negative affect was positively associated with the likelihood of NSSI urges or thoughts (see Table 3). In contrast, Kiekens et al. (2020) found an inverse relationship between positive affect and NSSI thoughts.

#### Lagged effects of affect on NSSIT

Seven studies (Dillon et al., 2021; Hepp et al., 2021; Hughes et al., 2019; Kiekens et al., 2020; Kranzler, 2016; Kranzler et al., 2018; Victor et al., 2019) examined lagged effects of negative emotion and NSSIT, whereby negative affect at one time‐point predicted NSSIT at a subsequent time point. However, two of these were based on the same sample (Hughes et al., 2019; Kranzler et al., 2018). Three of these studies measures urges rather than NSSI thoughts (Dillon et al., 2021; Hepp et al., 2021; Victor et al., 2019). Across these studies, greater negative affect was positively, significantly associated with the presence or intensity of NSSIT at a subsequent assessment point (Hughes et al., 2019; Kiekens et al., 2020; Kranzler, 2016; Kranzler et al., 2018), with the exception of Hepp et al. (2021). These associations were found in young adults and adolescents reporting a history of NSSIT. Relative risk values for subsequent NSSIT intensity were *RR* = 1.01–1.02, so that a point‐increase in negative affect was associated with a 10% or 20% increase in NSSIT intensity. Dillon et al. (2021) focused on feelings of anger/hostility in veterans, reporting significant lagged association with the occurrence of NSSI urges.

One study distinguished between internalising and externalising forms of negative affect, reporting that only internalising negative affect was a predictor of later NSSI urges, at the within‐person level (standardised *β* = .24; Victor et al., 2019). Internalising negative affect included fear, shame, and sadness, whereas, externalising negative affect referred to hostility, anger, and irritability (Victor et al., 2019). One paper reported that the specific emotions of anxiety and feeling overwhelmed predicted greater NSSIT intensity at the subsequent assessment (*RR* = 1.05 for both emotions; Hughes et al., 2019). Across all of these studies, sample sizes were small (*n* = 24–62). The presence of rumination increased the association between feeling overwhelmed, and subsequent NSSIT intensity (this interaction became non‐significant adjusting for gender) but reduced the effect of anxiety on NSSIT (to the point that the association between anxiety and NSSIT changed direction).

Three studies investigated positive affect and NSSIT. Two reported that greater positive emotion significantly predicted lower intensity of NSSIT at the subsequent assessment point (Kranzler, 2016; Kranzler et al., 2018). Kiekens et al. (2020) similarly reported that greater than usual positive affect (for that person) predicted a lower risk of NSSI thoughts, but this effect disappeared when adjusting for negative affect, and perceived self‐efficacy to resist NSSI.

#### Non‐linear changes in affect around episodes of NSSIT

Two study modelled the change in affect occurring over time around instances of NSSI urges in adults with a diagnosis of BPD (Hepp et al., 2021; Snir et al., 2015). They both reported a non‐linear pattern fitted the data whereby negative affect increased prior to and declined following the NSSI urge. In Snir et al. (2015), however, the rise in negative affect continued to a peak between 2–5 h following the urge, and then decreased quite sharply, rather than declining immediately after the urge was experienced.

### Nonsuicidal Self‐Injurious Behaviours (NSSI)

#### Concurrent associations of affect with NSSI

Four studies (Ammerman et al., 2017; Anestis et al., 2012; Selby et al., 2013; Vansteelandt et al., 2017) analysed concurrent associations between negative affect and NSSI. One study reported no significant association between general negative affect and frequency and/or occurrence of NSSI (Ammerman et al., 2017). In a sample of adults diagnosed with BPD (Vansteelandt et al., 2017), the average affect valence (pleasantness to unpleasantness), but not activation (i.e. more active or energised affective states) over the ESM period was lower in those who engaged in NSSI in this time by 85 points (the scale ranged from −200 to 200; between‐persons effect).

In one large model adjusting for multiple different variables (see Table 3; Briones‐Buixassa et al., 2021) feelings of frustration (OR = 1.05), but not guilt or sadness, were related to a greater likelihood of NSSI. In contrast to other studies, anger was related to a lower risk of NSSI (OR = 0.92). There were also multiple significant interaction effects, but these presented an inconsistent picture. Greater ability to decentre from undesirable internal states was related to a reduced effect of sadness on NSSI but led to a more positive relationship between anger and NSSI. Greater affective symptoms also led to a more positive relationship between anger and NSSI but led to a reduced association between frustration and NSSI. There was no testing of simple slopes which limits interpretation.

Three studies focused specifically on levels of instability in affect, creating aggregated indices of affective instability over time, and investigating the association this had with NSSI within the ESM period. Vansteelandt et al. (2017) reported an effect whereby greater frequency of NSSI during the ESM period was initially associated with a spike in within‐person variance in affect (degree of reported pleasure or displeasure) followed by a decline in variance, which levelled off at higher frequencies of NSSI. The authors suggest this pattern captures how greater use of NSSI may help stabilise affect, potentially illustrating one way NSSI may help individuals to cope. A further study (Selby et al., 2013) reported that NSSI risk was lower when both negative affect and rumination was stable, when compared with either or both these variables was unstable. When specific emotions were examined, a combination of greater instability in sadness and rumination was associated with the highest frequency of daily NSSI. In contrast, Anestis et al. (2012) reported no significant association between state affective instability on the occurrence of NSSI. This study aggregated across the ESM period and so overlooked potentially important within‐person variability in NSSI.

#### Lagged effects of affect on NSSI

Six studies (at least two sharing the same sample) examined lagged effects of negative affect and NSSI (Dillon et al., 2021; Houben et al., 2017; Hughes et al., 2019; Kiekens et al., 2020; Kranzler, 2016; Kranzler et al., 2018 ), consistently reporting that higher levels of negative affect at one time‐point predicted a higher probability or frequency of NSSI occurring at the subsequent assessment point. This finding was apparent across varied samples including adolescents and young adults reporting a history of NSSI, and adults with a diagnosis of BPD, though sample sizes were small (*n* = 24–47). Dillon et al. (2021) reported that feelings of anger/hostility, in veterans in particular, had significant lagged association with the occurrence of NSSI. Kiekens et al. (2020) reported that a similar association held for a variety of specific negative emotions (anxiety, stress, sadness, hopelessness, insecurity, though not for irritation). Hughes et al. (2019) found significant interaction effects whereby a positive association between negative affect (or feeling overwhelmed, specifically) with subsequent NSSI was more pronounced in the presence of high rumination.

Three studies stated that there were no significant predictive effects of positive emotion on the probability of engaging in NSSI in the next time interval (Houben et al., 2017; Kranzler, 2016; Kranzler et al., 2018). However, sample sizes were small (*n* = 24–47). Kiekens et al. (2020), in contrast, found that greater than usual positive affect was predictive of lower NSSI risk, and this effect held when investigating specific positive emotions (e.g. cheerful, satisfied).

#### Lagged effects of NSSI on affect

One study (Houben et al., 2017) examined the “reverse effect” of the studies above, analysing changes in negative emotion following acts of NSSI, and found that NSSI predicted a small increase in negative affect (*B* = 6.08, *p* < .01, negative affect was measured on a 100‐point scale) at the subsequent time interval. In contrast, positive affect showed a small decline (*B* = −5.82, *p* < .01, positive affect measured on a 100‐point scale). This result is inconsistent with other studies (see below) that demonstrate a decline in negative affect following NSSI. The authors acknowledged the potential limitations of their small sample size, and the possibility that their study design may not have been able to capture some initial emotional relief on a very short timescale (i.e. seconds and minutes) following NSSI. The study also adjusted for the outcome at the previous assessment point within lagged analyses.

#### Changes in affect around episodes of NSSI

Five studies (Andrewes et al., 2017a; Andrewes et al., 2017b; Armey et al., 2011; Muehlenkamp et al., 2009; Snir et al., 2015) examined changes in general negative affect occurring over time around episodes of NSSI. Three studies, two with larger samples (*n* > 100, though not all reported engaging in NSSI during ESM) found that these changes fit a quadratic pattern whereby negative affect increased prior to and decreased following NSSI episodes. This pattern was observed for the total number of negative emotions experienced (Andrewes et al., 2017b) and for specific emotional states, including guilt and anger (Armey et al., 2011). In one study, changes in negative affect occurred a median of 15.18 h before engaging in NSSI (Andrewes et al., 2017a). These patterns were found in a sample of adolescents and young adults with a diagnosis of first presentation BPD. Andrewes et al. (2017b) graphed these effects indicating that negative affect ratings peaked above 3 (on a 1 to 5 scale) when NSSI occurred, relative to scores below 2 for non‐NSSI comparators. This study also investigated changes in the experience of conflicting emotions (i.e. the co‐occurrence of two differently valenced emotions at the same time) but did not identify the same quadratic effect linked to NSSI.

Only one study (Snir et al., 2015) reported that there was no significant change in general negative affect surrounding NSSI acts (*n* = 99, analysis conducted on a smaller subset of participants). A further paper (Muehlenkamp et al., 2009) stated that although there was a significant increase in negative affect prior to NSSI in a sample of females with a diagnosis of BN, negative affect remained unchanged following NSSI acts. Various differences in study methodology could account for these inconsistent results. The Snir et al. (2015) study was the only one to also include individuals diagnosed with APD, and also used a non‐validated set of affect items developed for the study; whereas other studies used items from the Positive and Negative Affect Schedule (PANAS; Watson et al., 1988).

Two small studies (*n* = 24–47; Kranzler, 2016; Kranzler et al., 2018) focused on specific negative emotion in samples of adolescents and young adults with a history of self‐injury. They reported that feeling angry, hurt/rejected, frustrated, anxious/afraid, and overwhelmed decreased following NSSI (*d* = −.88 to .37), whereas there was no change in feelings of guilt, shame, feeling empty/numb, or embarrassed. There were some differences in findings between the two studies; one found that the feelings of sadness and loneliness did significantly decrease following an episode of NSSI (Kranzler et al., 2018) whereas the other found that they did not (Kranzler, 2016). Guilt was also shown to increase following NSSI in one study but not the other.

Five studies (Andrewes et al., 2017a; Armey et al., 2011; Kranzler, 2016; Kranzler et al., 2018; Muehlenkamp et al., 2009) examined changes in positive affect surrounding episodes of NSSI. Two studies reported that positive affect decreased prior to and increased following NSSI, fitting a quadratic pattern (Andrewes et al., 2017a; Muehlenkamp et al., 2009). In one of these studies, with a sample of adolescents and young adults with first presentation of BPD (Andrewes et al., 2017a), changes in positive affect occurred a median of 10.04 h before engaging in NSSI.

One study (Armey et al., 2011), in a small sample of students with a history of NSSI (*n* = 36), observed a slightly different temporal pattern whereby positive affect actually increased prior to NSSI and continued to increase following NSSI. This might reflect how just considering or planning NSSI resulted in an initial increase in positive affect but given this is the only study to identify such a pattern, this suggestion is speculative. Two further studies (Kranzler, 2016; Kranzler et al., 2018), in small samples of adolescents and young adults with history of NSSI (*n* = 24–47), examined changes in specific positive emotions following NSSI, finding that whilst some emotions increased after NSSI (happy, content, proud, relieved, calm, and satisfied; *d* = .49–1.51), others did not (“experiencing a rush or a high”, feeling excited).

#### NSSI urges and behaviour

One study, with a sample of 38 individuals with a diagnosis of BPD, assessed negative emotion differentiation, which is the ability to differentiate broad, negative emotional experience into more nuanced emotional categories (Zaki et al., 2013). Variables were aggregated across the ESM period allowing for a single‐level analysis, and NSSI urges and acts were combined into a single outcome variable. A significant interaction between rumination and negative emotion differentiation was reported, whereby a combination of high levels of rumination and low differentiation of negative emotion was associated with significantly increased frequency of NSSI urges. The aggregation of NSSI urges and acts, however, ignores potential differences in these phenomena, and the aggregation of data across the ESM period means that potentially important within‐person variance in scores was ignored.

## DISCUSSION

The aim of the current review was to summarise and critically evaluate existing ESM research regarding momentary emotional states and NSSIT and/or NSSI. Negative affect was generally found to be associated with both NSSIT and NSSI. The data supports a pattern whereby negative affect is higher prior to NSSI‐related behaviour and thoughts, and typically decreases following NSSIT and engagement in NSSI. This was apparent for general negative affect but also for a number of specific emotional states (e.g., anxiety, feeling overwhelmed, anger, hurt/rejected, frustrated) although evidence of this pattern was inconsistent or lacking for other emotional states (e.g., sadness, loneliness, embarrassed, ashamed, guilt). Results for positive affect were less consistent than for negative affect, possibly due to the smaller number of studies available. There was some evidence that positive affect decreased prior to and increased following episodes of NSSI, but other studies failed to find significant lagged associations between positive emotion and engagement in NSSI at the next time interval. With regards to NSSIT, greater positive emotion was found to be a significant predictor of lower intensity of NSSIT at the subsequent assessment point. There was evidence from two studies that some specific emotions increased following NSSI (e.g. happiness, content, proud, relieved, calm, satisfied). There was also evidence that affect may interact with rumination in predicting NSSI, though the small number of studies means these results are preliminary. Results regarding instability in affect were also mixed, with one study finding no significant association between state affective lability and number of NSSI episodes, but two others reporting that higher instability was predictive of NSSI, either as a main effect, or when moderated by instability in rumination.

Overall, the results are consistent with research highlighting that affect regulation, in particular the regulation of aversive emotional states, is a primary function of NSSI (Klonsky et al., 2014; Taylor et al., 2018). This is consistent with theoretical models of NSSI, which propose that the regulation of aversive emotional states is a major factor involved in the onset and maintenance of NSSI (e.g. Chapman et al., 2006; Hasking et al., 2017; Selby & Joiner, 2009). There was also preliminary evidence that other cognitive processes, namely rumination, may interact with affective experiences to predict NSSI thoughts and behaviour, as proposed by the Emotional Cascade Model (Selby & Joiner, 2009). However, effects seem to differ depending on the type of affective experience (e.g. differing effect for overall negative affect and specific emotions), and further replication of these effects is needed.

Mixed results regarding the associations between affect instability and NSSI are somewhat in contrast with previous studies, which suggest affective instability is an important correlate or predictor of NSSI (e.g. Peters et al., 2016; Santangelo et al., 2017). This could be due to the small sample size and potential low power of the study that did not report a significant effect. There was also inconsistency in the approach taken to creating a measure of affect instability and in how NSSI was modelled (e.g. as an in‐the‐moment experience, or a daily aggregate), which potentially contributed to inconsistency.

There were limitations within this literature. Many of the included articles used a small sample size, and none justified their sample size, which may have led to decreased power to detect effects and consequently increased Type II errors (false negatives). This problem may have been exacerbated by missing data in some studies. Despite this, studies often found significant effects which, given the small samples and lower power, could be indicative of publication bias within the field. Included studies did have a mix of clinical, non‐clinical, and mixed samples. However, the sampling procedures used mean that results may not be a true representation of the sample, due to the potential for self‐selection bias, and may not generalise to the wider populations of interest. The majority of studies focused on young adult samples, with only five studies having an average sample age over 25 years. There was no clear suggestion of age‐related differences in results, but the lack of variation in age, and general sample heterogeneity, made any such patterns hard to identify. Future work using older samples would be beneficial.

A lack of a validated measure for assessing NSSI during ESM was common. Many of the momentary NSSI items used by studies in this review have face validity, and are similar existing single‐item measures of NSSI (e.g. “Please indicate whether you injured yourself directly since the last diary”; Zaki et al., 2013). Nonetheless, it cannot be assumed that measures with sound psychometric properties, as established in traditional questionnaire settings, can simply be administered, or administered with small adaptions, in an ESM context without implications for their reliability and validity. However, this seems to be an issue across non‐ESM NSSI literature also (Robinson & Wilson, 2020). Despite the use of non‐validated items being common, we recommend that future research makes use of the Experience Sampling Item Repository (Kirtley et al., 2021), which is an ongoing open science project that aims to produce an open bank of ESM items and to quality assess and psychometrically validate these items.

All but one of the articles included in this paper were sourced from peer‐review journals (with one being a dissertation), and although the researcher contacted authors for any unpublished data, other eligible research in the grey literature may not have been included in this review. Furthermore, this review only included papers that were written in English, leading to a similar issue of otherwise eligible research being excluded, though only one paper was excluded at the full‐text stage on this basis.

Studies with larger sample sizes and broader sampling methods are needed to reduce the risk of Type II errors. Pooling of results could also be beneficial for this reason, and to provide more reliable and precise results. However, aggregation of data fundamentally depends on clear and explicit reporting of study characteristics, as well as the use of validated, standardised measures of key constructs. Studies should be pre‐registered to reduce publication and reporting bias, and authors should ensure their work adhere, where possible, to ESM reporting guidelines (Trull & Ebner‐Priemer, 2020). Studies should be pre‐registered to reduce publication and reporting bias. Missing data is often an issue within ESM studies due to the nature of the methodology (Carter & Emsley, 2019). This could be improved by reflecting on ways to increase engagement and compliance with the methodology, and also by allowing event‐contingent entries. Future studies could also consider combining ESM with qualitative methodology in order to broaden information gleaned from in‐the‐moment sampling.

Overall, the results are largely consistent with the idea that NSSI may be a response to changes in negative affect and may operate to regulate or reduce these emotions. These results support the use of therapies that target emotion regulation difficulties, such as dialectical behaviour therapy (DBT), which has been found to be effective for emotion regulation and NSSI (Gibson et al., 2014; Linehan et al., 2009; Mehlum et al., 2014; Mehlum et al., 2016; Mehlum et al., 2019). Interventions that help individuals to better recognise momentary changes in affect and develop alternative responses than NSSI (this may include both intra and interpersonal strategies), may be helpful. ESM could also be used as part of therapy, as opposed to traditional pen and paper tracking diaries, as this could provide fine‐grained in‐the‐moment information surrounding NSSI and emotional states (e.g., internal and situational determinants), that are less subject to reporting biases that may influence responses to traditional symptom measures. ESM‐based interventions may aid individuals in monitoring affective states and help facilitate them in applying techniques (e.g. distraction or coping techniques) in the moment. There is preliminary evidence that such interventions may be helpful (Arshad et al., 2019).

### AUTHOR CONTRIBUTION

**Adam Danquah** (Conceptualization; Writing – review & editing) **Peter Taylor** (Conceptualization; Data curation; Formal analysis; Methodology; Project administration; Supervision; Writing – review & editing) **Terence Brown** (Formal analysis) **Alexandra Brown**, ClinPsyD (Conceptualization; Data curation; Formal analysis; Project administration; Writing – original draft; Writing – review & editing) **Katie Dhingra** (Formal analysis; Writing – review & editing).

### CONFLICT OF INTEREST

No conflict of interest.

## Supporting information


Appendix S1

## Data Availability

Data sharing is not applicable to this article as no new data were created or analysed in this study.

## Footnotes

^1^
The term Ecological Momentary Assessment (EMA) is more commonly used in the US, whilst the term Experience Sampling Methodology (ESM) is more commonly used in the UK and Europe. We use the terms ESM here given the team were UK based and more familiar with this phrasing.
