# Supplementary material for: A systematic review of the relationship between momentary emotional states and nonsuicidal self‐injurious thoughts and behaviours
Source: Psychol Psychother. 2022 May 8;95(3):754–80. doi: 10.1111/papt.12397 (PMC9833836; doi:10.1111/papt.12397)
Supplement: Supplementary file 1 — Appendix S1 [file PAPT-95-754-s001.docx]

**Supplement I**

**Departures from Protocol**

Within the original protocol the review was planned to include suicidal behaviours as well as Non-Suicidal Self-Injury. The review aims were later revised to focus specifically on NSSI. This change occurred during the process of screening and was based on the following considerations:

1. The majority of identified Experience Sampling Methodology (ESM) appeared to focus on NSSI, rather than suicidal behaviour.
2. Focussing on just NSSI felt beneficial in streamlining the review and increasing the focus.
3. On reflection the research team decided that trying to encompass both studies about NSSI and suicidal behaviour would be difficult within a single review, and the ESM literature concerning suicidal thoughts and behaviours would be better served by a separate review.

**Supplement II**

**MESH Terms used in Database Searches**

In addition to the keywords used in searches of electronic databases, the following Medical Subject Heading (MESH) terms were also used

| Database | MESH terms |
| --- | --- |
| PsycInfo | **Self-Destructive Behaviour**  **Self-Injurious Behaviour Self-Inflicted Wounds**  **Self-Mutilation**  **Emotions**  **Positive Emotions**  **Negative Emotions** |
| MEDLINE | **Self-injurious Behaviour** **Emotions**  **Affect** |

**Supplement III**

**Risk of Bias of observational studies**

Risk of bias was assessed using a tool adapted from the Agency for Healthcare Research and Quality (Williams et al., 2010), which has been used in previous systematic reviews of observational research in the area of NSSI (Taylor et al., 2015; Taylor et al., 2018). The tool is presented below. As the tool was not developed for ESM studies in particular, three additional domains were added that reflected key methodological features in ESM research. These additional domains were developed by the research team based on existing best practice guidance concerning ESM, including:

Palmier-Claus, J., Haddock, G., & Varese, F. (2019.), *Experience sampling in mental health research*. New York: Routledge.

Palmier-Claus, J. E., Myin-Germeys, I., Barkus, E., Bentley, L., Udachina, A., Delespaul P. A .E. G., Lewis, S. W., & Dunn, G. (2011). Experience sampling research in individuals with mental illness: Reflections and guidance. *Acta Psychiatrica Scandinavica, 123*, 12-20. https://doi.org/10.1111/j.1600-0447.2010.01596.x

These additional items included the use of a method that allows assessments to be time stamped (e.g. completion of ESM assessments through an electronic device rather than a paper diary), the use of random or pseudo-random timing of assessment prompts (as opposed to assessments at fixed times of day), and the removal of assessments from analysis where the response fell outside a 15 minute window surrounding the prompt. A strength of ESM is seen to be the capture of spontaneous and in-the-moment experience, and hence the ability to time-stamp responses (to prevent retrospective reporting or back-filling of assessments), and the exclusion of data points that occur to far from an initial prompt was given, is seen as important. Likewise the use of random or pseudo-random prompts is seen as important in ensuring participants do not anticipate and prepare for assessments in advance, and to minimise any effects related to specific times of the day when prompts occur.

**General instructions:** Grade each criterion as “Yes,” “No,” “Partially,” or “Can’t tell.” Factors to consider when making an assessment are listed under each criterion. Note that some criteria will only apply to specify types of study. For example, power calculations are relevant for studies aiming to compare suicide risk between two groups, or studies that look at correlates of study outcomes. Where a criterion only applies to a specific design, it is in italics. The examples given for studies meeting, or not meeting, criterion may not apply in every instance. The review team should carefully consider any adaptations needed to this tool and ensure these are implemented prior to starting the Risk of Bias assessment.

| **Criteria** | **Yes - criteria met** | **No - criteria not met** |
| --- | --- | --- |
| **Unbiased selection of the cohort?**  To consider: is the sample representative of the population of interest?  What is the risk of self-selection bias? | - True random sample or method that approximates this (e.g. stratified cluster sampling). - All potentially eligible consecutive referrals at a service or clinic are invited to take part in the study - All patients at a service or students within a University are invited - In this case potential participants still have the option to say no and not participate, and so self-selection bias is introduced, but the means of identifying and approaching potential participants does not impose further risk of self-selection. | - Method of sampling liable to introduce substantive self-selection bias - Snowball sampling - Advertising placed in selected locations (e.g. waiting rooms, around University campus) - Advertising via social media   **CONSIDER PARTIAL RATING IF:**  Recruitment methods above are used, where self-selection bias likely, but a wider range of recruitment sites or sources are used (e.g. social media and clinical services and community groups) so that the impact of self-selection might be limited. |
| **Sample size calculated?** | - Sample size is justified with power calculation, simulation or other appropriate method - Eventual sample size does not deviate by > 10% from the sample size suggested | - No justification of sample size is given   **CONSIDER PARTIAL RATING IF:**  Any justification of sample size provided, but not a power calculation |
| **Adequate description of the cohort?** | - Its clear what population participants come from - Age and gender are reported - Ethnicity reported or other information concerning participants’ demographic background such as education, employment or socio-economic status is given **(may be partial rating if this is missing).** | - Sample age and gender not reported |
| **Validated method for ascertaining clinical status or participant group**  **Note: this also includes samples with a common, clinically relevant, status, such as survivors of sexual abuse**  To consider: What is the risk of individuals being incorrectly identified (false positives and negatives) | - Validated instrument used to determine relevant clinical status - Valid method of ascertaining diagnosis or clinical ‘caseness’ (e.g., clinical interview) | - This will depend on researchers’ discretion over what constitutes a valid method of ascertaining this information, but non-valid methods may include: - Self-report (or self-report when not obtained through a validated assessment tool) - Chart diagnoses or reliance on medical notes not otherwise confirmed by researchers |
| **Validated methods for assessing emotion during ESM** | - Measures used have been previously validated in other research with evidence of acceptable reliability and validity   **CONSIDER PARTIAL RATING IF:**  The measure has previously been validated but in the current study sample has poorer psychometric properties such as an internal reliability *ɑ* < .6 | - Tool or measure developed specifically for the study - No psychometric evaluation undertaken, or very minimal evaluation (e.g. internal consistency only)   **CONSIDER PARTIAL RATING IF:**  The measure has not previously been validated but in the current study sample good evidence of psychometric properties is shown, such as good reliability or results of factor analysis. |
| **Validated methods for assessing NSSI/NSSIT during ESM** | - Measures used that has been previously validated in other research with evidence of acceptable reliability and validity - Other valid process for determining outcome may include clinical diagnosis or coroner reports (e.g. if outcome is suicide)   **CONSIDER PARTIAL RATING IF:**  The measure has previously been validated but in the current study sample has poorer psychometric properties such as an internal reliability below .6 | - Tool or measure developed specifically for the study - No psychometric evaluation undertaken, or very minimal evaluation (e.g. internal consistency only)   **CONSIDER PARTIAL RATING IF:**  The measure has not previously been validated but in the current study sample good evidence of psychometric properties is shown, such as good reliability or results of factor analysis. |
| **Missing data is minimal** | - Missing ESM data does not exceed 30% (Either this is explicitly indicated or highly likely based on available information about compliance). | - Missing ESM data exceeds 40% or is not suitably managed.   **CONSIDER PARTIAL RATING IF:**  The missing ESM data is between 30-40% |
| **Analysis controls for confounding variables** | - Conceptually relevant covariates (potential confounding variables) are adjusted for within analyses. | - No covariates are adjusted for within analyses. |
| **Analytic methods appropriate** | - Analysis was appropriate given the type of data (categorical, continuous, etc.), and type of association being tested. - Analysis takes into account issues such as clustering, rare outcomes, multiple comparisons, etc. | - Analysis was not suitable given the type of data or type of associations being tested   **CONSIDER PARTIAL RATING IF:**   - Appropriate analytic method used but analysis controls for prior instances of the outcome as a covariate |
| **Time-stamped response** | - ESM responses are time-stamped by electronic system | - ESM responses are not time stamped by electronic system |
| **Pseudo-random prompts** | - ESM prompts are issued at random or pseudorandom times | - ESM prompts are not issued at pseudorandom times |
| **Included timepoints completed within 15 minutes** | - ESM entries included for analysis are completed within 15 minutes of prompt | - ESM entries occurring after 15 minutes of the prompt included in analysis |
